# Associations between sleep disturbances, diabetes and mortality in the UK Biobank cohort: A prospective population‐based study

**DOI:** 10.1111/jsr.13392

**Published:** 2021-06-08

**Authors:** Malcolm von Schantz, Jason C. Ong, Kristen L. Knutson

**Affiliations:** ^1^ Faculty of Health and Medical Sciences University of Surrey Guildford UK; ^2^ Department of Neurology Center for Circadian and Sleep Medicine Northwestern University Chicago IL USA

**Keywords:** cohort study, diabetes mellitus, insomnia, non‐communicable disease, sleep disorders

## Abstract

Non‐communicable diseases, including diabetes, are partly responsible for the deceleration of improvements of life expectancy in many countries. Diabetes is also associated with sleep disturbances. Our aim was to determine whether sleep disturbances, particularly in people with diabetes, were associated with increased mortality risk. Data from the UK Biobank were analysed (*n* = 487,728, mean follow‐up time = 8.9 years). The primary exposure was sleep disturbances, assessed through the question: Do you have trouble falling asleep at night or do you wake up in the middle of the night? The primary outcome was mortality. We also dichotomized sleep disturbances into “never/sometimes” versus “usually” (frequently), and combined with the presence/absence of diabetes: 24.2% of participants reported “never/rarely” experiencing sleep disturbances, 47.8% “sometimes” and 28.0% “usually”. In age‐ and sex‐adjusted models, frequent sleep disturbances were associated with an increased risk of all‐cause mortality (hazard ratio [HR], 1.31; 95% confidence interval [CI], 1.26–1.37), which remained significant in the fully adjusted model (HR 1.13, 95% CI 1.09–1.18). The presence of both diabetes and frequent sleep disturbances was associated with greater risk of all‐cause mortality than either condition alone. In the fully adjusted model, the hazard ratio for all‐cause mortality was 1.11 (95% CI, 1.07–1.15) for frequent sleep disturbances alone, 1.67 (95% CI, 1.57–1.76) for diabetes alone and 1.87 for both (95% CI, 1.75–2.01). Frequent sleep disturbances (experienced by more than one quarter of the sample) were associated with increased risk of all‐cause mortality. Mortality risk was highest in those with both diabetes and frequent sleep disturbances. Complaints of difficulty falling or staying asleep merit attention by physicians.

## 1 INTRODUCTION

Increases in life expectancy have slowed or even ceased in the United States, the United Kingdom and comparable countries (Murphy, Xu, Kochanek, & Arias, 2017; Office for National Statistics, 2018). In the majority of countries, people have a high risk of premature mortality due to non‐communicable diseases (NCDs), including diabetes, compared with other conditions (NCD Countdown 2030 Collaborators, 2018). The mortality rates due to these NCDs are expected to increase by approximately 54% between 2016 and 2040, and deaths due specifically to type 2 diabetes are estimated to more than double worldwide (Foreman, Marquez, & Dolgert, 2018). Indeed, diabetes increases the risk of all‐cause and cardiovascular mortality (Haffner, Lehto, Ronnemaa, Pyorala, & Laakso, 1998). An international group led by the United Nations set a goal of reducing the rates of premature mortality due to NCD by one third by the year 2030, but only 16% of countries are on target for men and 19% of countries are on target for women (NCD Countdown 2030 Collaborators, 2018). Given that NCDs are the leading causes of death and the rates of premature mortality rates are not declining as targeted, it is important to gain a greater understanding of the underlying causes of NCD‐associated mortality. Here, we have used data from the UK Biobank to examine the effect of sleep disturbances and their interactions with diabetes, a major NCD, on morbidity and mortality.

Diabetes and early mortality have both been associated with inadequate sleep, including insufficient duration or poor quality of sleep (e.g., Akerstedt et al., 2017; Anothaisintawee, Reutrakul, Van Cauter, & Thakkinstian, 2016; Heslop, Smith, Metcalfe, Macleod, & Hart, 2002; Hublin, Partinen, Koskenvuo, & Kaprio, 2007; Kripke, Langer, Elliott, Klauber, & Rex, 2011; Tamakoshi & Ohno, 2004). A number of studies have examined sleep disturbances or insomnia complaints in relation to mortality risk, and some observed significantly increased risk of mortality associated with these sleep‐related complaints (Li et al., 2014; Sivertsen et al., 2014); however, others did not (Althuis, Fredman, Langenberg, & Magaziner, 1998; Kripke, Garfinkel, Wingard, Klauber, & Marler, 2002; Lovato & Lack, 2019; Rockwood, Davis, Merry, Macknight, & Mcdowell, 2001). This discrepancy could be due to differences in the demographics of the sample, as ages and proportions of men and women varied among studies, as did sociodemographic and cultural factors. People with diabetes generally have poorer sleep quality (Trento et al., 2008) and poorer sleep quality has been associated with worse glycaemic control (Knutson, Ryden, Mander, & Van Cauter, 2006; Knutson, Van Cauter, Zee, Liu, & Lauderdale, 2011). Whether the combination of diabetes and frequent sleep disturbances affects mortality risk has not been previously reported.

The aims of the analyses presented here were to determine whether sleep disturbances were associated with increased risk of all‐cause and cardiovascular disease (CVD) mortality in a large study of adults in the UK and to determine whether having both frequent sleep disturbances and diabetes was more strongly associated with mortality than either condition on its own. We hypothesized that frequent sleep disturbances would be associated with increased mortality risk, particularly in those with diabetes.

## 2 METHODS

We used data from the UK Biobank, which is a large, prospective, population‐based cohort study designed to investigate risk factors for major diseases of middle and older age (Sudlow et al., 2015). It enrolled 502,642 people aged 37–73 years (53% women) from across the UK between March 2006 and October 2010. The study invited every individual within this age range who was registered with the National Health Service and living up to about 25 miles from an assessment centre to participate (Allen et al., 2012). Identical assessment procedures were used across field sites. For the analyses presented here, we have death records up to 14 February 2018, which resulted in a mean follow‐up time of 8.9 years (range, 4 days to 11.9 years) among participants and that 98% of deaths occurred after 6 months of follow‐up. The UK Biobank protocol is available online (Palmer, 2007).

The primary exposure variable was the presence of sleep disturbances, as assessed through a single question that asked: Do you have trouble falling asleep at night or do you wake up in the middle of the night? There were three response options: “never/rarely”, “sometimes” or “usually”. Only 0.1% of participants did not answer this question.

The primary outcome was mortality, including all‐cause mortality and mortality due to CVD. Mortality information was obtained from the National Health Service for England and Wales and the NHS Central Register in Scotland. All details from the death certificates were provided to UK Biobank personnel, who coded primary cause of death according to ICD10. We classified the ICD10 codes I00–I99 as CVD‐related mortality. If no death was recorded for a participant, they were assumed to still be living.

We examined the interaction between the presence of sleep disturbances and the presence of diabetes. Participants were classified as having diabetes if they reported a previous diagnosis of diabetes or they reported taking insulin. We dichotomized sleep disturbances into “never”/”sometimes” (infrequently) versus “usually” (frequently) and combined this with presence/absence of diabetes to create four groups. We also created six groups based on three levels of frequency of sleep disturbance and presence of diabetes.

Covariates included age, sex, ethnicity, socioeconomic deprivation, smoking, sleep duration, body mass index (BMI) and comorbidities. Age was calculated based on date of birth and date of examination. Sex was acquired from the central registry and updated by the participant, if needed. Ethnicity was self‐identified and the majority of the sample (94%) described themselves as “white”. Therefore, we dichotomized ethnicity into “white” and “non‐white”. The measure of socioeconomic deprivation was based on the Townsend deprivation index (Townsend et al., 1988), which summarizes deprivation in a postcode area based on the rates of unemployment, absence of ownership of a car and home, and household overcrowding. Smoking status was obtained by self‐report with the following categories: “never”, “previous smoker”, “current smoker” and “prefer not to answer”. Sleep duration was based on the question: About how many hours sleep do you get in every 24 h (please include naps)? Responses were provided as integers. Standing height was measured using a Seca 240‐cm height measure while participants stood barefoot with posture verified by trained staff. Weight was measured using a Tanita BC418MA body composition analyser and BMI (kg/m^2^) was calculated. Comorbidities were recorded based on self‐report during an interview by a trained nurse and all comorbidity variables are dichotomous (present/absent). We used the codes provided by the UK Biobank to classify them into the following 11 comorbidity variables for analyses (see Table S1 for complete list of codes): CVD, diabetes, other endocrine disorders, neurological disorders, renal disorders, respiratory disorders, musculoskeletal disorders, gastrointestinal/abdominal disorders, depression, other psychological disorders and cancer. A participant only needed a report of one code to be classified as having that comorbidity. Each comorbidity was treated as a separate variable in the analyses.

Insomnia symptom data were missing for 1504 participants, 9705 participants were missing BMI, 3000 were missing sleep duration, 603 were missing the Townsend index and three participants had a negative follow‐up period, resulting in a final sample size of 487,728 participants. Means and standard deviations (*SD*) or proportions (%) were calculated to describe the sample. Cox proportional hazards models were estimated to determine associations with all‐cause and CVD mortality. There were two models, one adjusting for age and sex only and one adjusting for all covariates. Further, we examined sleep disturbances alone (“never/rarely” was referent) as well as the four or six groups based on frequency of sleep disturbances and diabetes (absence of both was referent). In addition, to determine whether the two groups with diabetes differed, we repeated the analysis with the four groups using the diabetes‐alone group as the referent. Finally, we tested an interaction term between diabetes and sleep disturbances to determine whether the association between sleep disturbances and mortality risk varied between those with and without diabetes. All statistical analyses were performed using Stata, v14 (Stata corp).

## 3 RESULTS

Table 1 describes the sample in full as well as stratified by sleep disturbances. Approximately one quarter of the sample reported “never/rarely” experiencing sleep disturbances, 47.8% answered “sometimes” and 28.0% answered “usually”. On average, those who reported frequent sleep disturbances were older, had a higher BMI, slept less, and were more likely to be female, white, current smokers, and have depression and diabetes. Approximately 69% of the sample had neither diabetes nor frequent sleep disturbances, 26% had frequent sleep disturbances but not diabetes, 3% had diabetes but not frequent sleep disturbances and 2% had both. During the mean follow‐up period of 8.9 years, there were 19,177 deaths from all causes and 3,874 deaths from CVD.

**TABLE 1:** Description of full sample and by insomnia symptom frequency

	Full sample	Sleep disturbances		
		Never/rarely	Sometimes	Usually
*N*	487,728	118,217 (24.2%)	233,177 (47.8%)	136,334 (28.0%)
Age (years)^a^	56.5 (8.1)	55.2 (8.4)	56.6 (8.1)	57.5 (7.7)
Female^a^	54.4%	42.5%	56.3%	61.7%
White ethnicity^a^	94.4%	93.6%	94.2%	95.5%
Smoking status^a^				
Never	54.6%	57.1%	55.4%	51.1%
Previous	34.6%	32.3%	34.1%	37.3%
Current	10.5%	10.3%	10.1%	11.3%
Depression^a^	5.6%	3.3%	5.2%	8.2%
Diabetes^a^	5.0%	4.4%	4.6%	6.3%
BMI (kg/m^2^)^a^	27.4 (4.8)	27.2 (4.5)	27.3 (4.7)	27.9 (5.1)
Sleep duration (h)^a^	7.2 (1.1)	7.4 (1.0)	7.3 (1.0)	6.7 (1.3)

Note

Mean (standard deviation) for continuous variables; % for categorical.

Abbreviation: BMI, body mass index.

^a^ *p* < 0.01 comparing insomnia symptom groups based on ANOVA (continuous variables) or chi squared (categorical variables).

**FIGURE 1:**
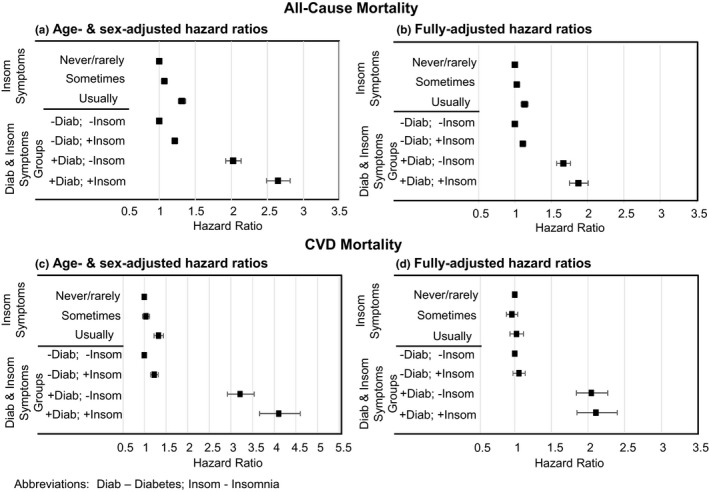
Hazard ratios (95% confidence interval [CI]) for mortality for sleep disturbances and for diabetes (Diab) and insomnia (Insom) groups. (a) Age‐ and sex‐adjusted hazard ratios for all‐cause mortality. (b) Fully adjusted hazard ratios for all‐cause mortality. (c) Age‐ and sex‐adjusted hazard ratios for cardiovascular disease (CVD) mortality. (d) Fully adjusted hazard ratios for CVD mortality. Fully‐adjusted models included the following covariates: age, sex, race, smoking status, body mass index (BMI), sleep duration, Townsend deprivation index, and all comorbidities (CVD, diabetes, other endocrine disorders, neurological disorders, renal disorders, respiratory disorders, musculoskeletal disorders, gastrointestinal disorders, depression, other psychological disorders and cancer)

Frequent sleep disturbances were significantly associated with all‐cause mortality (Figure 1). In age‐ and sex‐adjusted models, frequent sleep disturbances were associated with increased risk of all‐cause mortality (hazard ratio [HR], 1.31; 95% confidence interval [CI], 1.26–1.37), which remained significant in the fully adjusted model (HR. 1.13; 95% CI, 1.09–1.18). Frequent sleep disturbances were significantly associated with increased risk of CVD mortality in the age‐ and sex‐adjusted models (HR, 1.33; 95% CI, 1.22–1.44) but this association did not remain significant in the fully adjusted models (HR. 1.02; 95% CI, 0.93–1.12).

The analysis of sleep disturbances and diabetes demonstrated that the presence of either frequent sleep disturbances or diabetes was associated with increased risk of all‐cause mortality compared to those who had neither condition (Figure 1). In the fully adjusted model, the hazard ratio for all‐cause mortality was 1.11 (95% CI, 1.07–1.15) for frequent sleep disturbances alone, 1.67 (95% CI, 1.57–1.76) for diabetes alone and 1.87 for both (95% CI, 1.75–2.01). In addition, the group who had both frequent sleep disturbances and diabetes demonstrated a greater risk of all‐cause mortality compared to those with only diabetes (HR, 1.12; 95% CI, 1.04–1.22). Both groups with diabetes had an increased risk of CVD mortality compared to those with neither frequent sleep disturbances nor diabetes. In fully adjusted models, the HR was 2.04 (95% CI, 1.84–2.27) for diabetes alone and 2.11 (95% CI, 1.85–2.40) for both. There was no significant difference in risk of CVD mortality between the two diabetes groups (HR 1.03, 95% CI 0.89–1.20 for both frequent insomnia and diabetes compared to diabetes alone). The interaction between sleep disturbances and diabetes was not significant in either the model predicting all‐cause mortality (*p* = 0.40) or the model predicting CVD mortality (*p* = 0.68), which indicates the association between sleep disturbances and mortality did not differ between those with and without diabetes. In analyses that created six groups based on diabetes and three levels of sleep disturbances (Table S2), similar associations were observed. In the fully adjusted models, the group without diabetes but with frequent sleep disturbances and all three groups with diabetes had an increased risk of all‐cause mortality compared to the group with neither diabetes nor sleep disturbances.

## 4 DISCUSSION

In this large, UK‐based population study, we observed significant associations between frequent sleep disturbances and risk of all‐cause mortality. Frequent sleep disturbances were experienced by more than one quarter of the sample, and thus are highly prevalent in the UK, which is consistent with other observational studies (Ohayon, 2002). In addition, individuals with diabetes who also experienced frequent sleep disturbances had a greater risk of mortality than those with diabetes who did not report frequent sleep disturbances. Further, the association between frequent sleep disturbances and mortality risk did not differ between those who did and did not have diabetes.

Our findings from this UK cohort are consistent with studies from other countries. A study from Norway assessed self‐reported insomnia in adults aged 40–45 years at baseline and followed them for 13–15 years. They reported that insomnia at baseline was a significant predictor of all‐cause mortality (HR, 3.34; 95% CI, 1.67–6.69) (Sivertsen et al., 2014). A large study of middle‐aged and older men in the US also reported that difficulty initiating sleep (HR, 1.55; 95% CI, 1.19–2.04) and non‐restorative sleep (HR, 1.32; 95% CI, 1.02–1.72) were associated with increased risk of all‐cause mortality (Li et al., 2014). A Chinese study observed that adults who reported sleep disturbances nearly every day had an increased risk of mortality over approximately 16 years (Chien et al., 2010). A community‐based prospective study in the USA with a 20‐year follow‐up found that persistent insomnia (reporting symptoms at two assessments) was associated with increased mortality risk; however, reporting sleep disturbances at only one assessment was not (Parthasarathy, Vasquez, & Halonen, 2015). Not all studies that examined indicators of insomnia observed significant associations with mortality risk (Althuis et al., 1998; Kripke et al., 2002; Lovato & Lack, 2019; Rockwood et al., 2001). The discrepant findings could be due to differences in insomnia assessment, characteristics of the population studied or covariate adjustment, including comorbidities.

Sleep disturbances have been associated with CVD in prior research. For example, one prospective population‐based study from Norway followed participants for approximately 11 years and found that risk of acute myocardial infarction was significantly higher for individuals who had difficulty falling asleep almost every night (HR, 1.45; 95% CI, 1.18–1.80) and for individuals who had difficulties maintaining sleep almost every night (HR, 1.30; 95% CI, 1.01–1.68) compared to those who never have these sleep difficulties (Laugsand, Vatten, Platou, & Janszky, 2011). The same study also observed a significant increased risk of heart failure among those who had difficulty falling asleep almost every night (HR, 1.32; 95% CI, 1.01–1.72) compared to those who never have these sleep difficulties (Laugsand, Strand, Platou, Vatten, & Janszky, 2014). A large population‐based study in Taiwan also observed a significant increased risk of acute myocardial infarction, as well as stroke, among people with diagnosed insomnia (Hsu et al., 2015). In our study, however, we did not observe a significant association between frequent sleep disturbances and CVD mortality during the 8.9‐year follow‐up period. This may be because mortality from CVD is not impacted by sleep disturbances (at least as defined by the single question used here or limited power due to lower number of CVD deaths).

To our knowledge, this is the first study to examine the effect of the combination of insomnia and diabetes on mortality risk. Diabetes has been previously associated with increased risk of CVD and mortality (Haffner et al., 1998; Stamler, Vaccaro, Neaton, & Wentworth, 1993), and diabetes has been associated with impaired sleep. Several studies have found a strong association between obstructive sleep apnea (OSA) and type 2 diabetes (Huang et al., 2018; Subramanian et al., 2019), and OSA impairs sleep quality. Further, some observational studies have found that among people with type 2 diabetes, worse sleep quality is associated with higher haemoglobin A1c, suggesting poorer glycaemic control (Knutson et al., 2006, 2011). A meta‐analysis of nine studies among adults with type 2 diabetes also found that poor sleep quality was associated with higher haemoglobin A1c (Lee, Ng, & Chin, 2017), and insomnia has been identified as a risk factor for type 2 diabetes both in observational (Vgontzas et al., 2009) and Mendelian randomization studies (Yuan & Larsson, 2020). Experimental studies that impaired sleep quality did observe impairments in glucose regulation in healthy volunteers (Stamatakis & Punjabi, 2010; Tasali, Leproult, Ehrmann, & Van Cauter, 2008). If chronic poor sleep quality due to a sleep disorder can impair glucose control in people with diabetes, then this could be a mechanism leading to the increased risk of mortality in people with diabetes and frequent sleep disturbances.

The strengths of this study include the large sample size and the prospective monitoring of mortality. The UK Biobank study aimed to assemble a general population sample; however, this cohort does appear to be slightly healthier on average than the general UK population (Fry, Littlejohns, & Sudlow, 2017), which may limit generalizability somewhat. We also do not have access to measures of hypnotic or alcohol use in our dataset and these could be important confounders or mediators of the association between sleep disturbances and mortality. Another limitation is that the mean follow‐up time is only ​8.9 years and a longer period would result in a higher number of mortalities, which could increase power for the CVD mortality analyses. Finally, sleep disturbances are based on a single self‐reported question, which did not assess daytime consequences, and are not equivalent to a clinically diagnosed insomnia disorder. However, this same question in the same sample has recently been used successfully for genome‐wide association studies (Jansen, Watanabe, & Stringer, 2019; Lane et al., 2016), with the most significant hits being replicated both in a sub‐stratification of the UK Biobank study based on accelerometry and in a separate insomnia cohort (Lane et al., 2016). In addition, people who report sleep disturbances are likely to be a heterogeneous group with respect to the underlying pathology. Further, these data do not include objective measures of sleep quality, sleep duration or sleep‐disordered breathing so we cannot identify subtypes of insomnia or other sleep disorders. The data presented here suggest that regardless of the cause of the sleep disturbance, reporting sleep disturbances on a frequent basis is an important signal of an elevated risk of mortality. Such symptoms should therefore be investigated further by physicians, particularly in patients who have also been diagnosed with diabetes. Follow‐up instruments, such as the insomnia severity index (ISI), have the potential to further refine the understanding of the nature of the complaint and could feasibly be included in larger cohort studies.

Because a large proportion of the sample reported frequent sleep disturbances (28%), these findings have important public health implications. The results are also relevant to clinical practice and frequent sleep disturbances may be an important health indicator for clinicians to consider, particularly for diabetes patients. We found that a single question was sufficient to detect mortality risk and clinicians could use a similar brief question to identify patients who may need additional therapy or support.

## CONFLICT OF INTEREST

MvS, JCO and KLK: no conflict of interests.

## AUTHOR CONTRIBUTIONS

KLK and MvS designed the study, KLK analysed the data, and KLK, MvS and JCO wrote the paper.

## Supporting information

Table S1‐S2

## Data Availability

The data that support the findings of this study are available from the UK Biobank. Interested researchers should contact the UK Biobank for access to data. More information can be found here: https://www.ukbiobank.ac.uk/
