# Supplementary material for: Associations between sleep disturbances, diabetes and mortality in the UK Biobank cohort: A prospective population‐based study
Source: J Sleep Res. 2021 Jun 8;30(6):e13392. doi: 10.1111/jsr.13392 (PMC8612946; doi:10.1111/jsr.13392)
Supplement: Supplementary file 1 — Table S1‐S2 [file JSR-30-e13392-s001.docx]

**Online Supplemental Material**

Title: Associations between insomnia symptoms, diabetes and mortality in the UK Biobank cohort: a prospective population-based study

Authors: Malcolm von Schantz, Jason C. Ong, Kristen L. Knutson

This file includes two Supplemental Tables.

Supplementary Table 1. Specific codes used to identify the 11 comorbid conditions.

| Comorbidity Variable | UKB Variable | Code | Meaning |
| --- | --- | --- | --- |
| CVD | 20002 | 1065 | hypertension |
|  |  | 1066 | heart/cardiac problem |
|  |  | 1067 | peripheral vascular disease |
|  |  | 1068 | venous thromboembolic disease |
|  |  | 1072 | essential hypertension |
|  |  | 1073 | gestational hypertension/pre-eclampsia |
|  |  | 1074 | angina |
|  |  | 1075 | heart attack/myocardial infarction |
|  |  | 1076 | heart failure/pulmonary odema |
|  |  | 1077 | heart arrhythmia |
|  |  | 1078 | heart valve problem/heart murmur |
|  |  | 1079 | cardiomyopathy |
|  |  | 1080 | pericardial problem |
|  |  | 1081 | stroke |
|  |  | 1082 | transient ischaemic attack (tia) |
|  |  | 1083 | subdural haemorrhage/haematoma |
|  |  | 1086 | subarachnoid haemorrhage |
|  |  | 1087 | leg claudication/ intermittent claudication |
|  |  | 1088 | arterial embolism |
|  |  | 1093 | pulmonary embolism dvt |
|  |  | 1094 | deep venous thrombosis (dvt) |
|  |  | 1426 | myocarditis |
|  |  | 1471 | atrial fibrillation |
|  |  | 1473 | high cholesterol |
|  |  | 1483 | atrial flutter |
|  |  | 1484 | wolff parkinson white / wpw syndrome |
|  |  | 1485 | irregular heart beat |
|  |  | 1487 | svt / supraventricular tachycardia |
|  |  | 1488 | mitral valve prolapse |
|  |  | 1489 | mitral stenosis |
|  |  | 1490 | aortic stenosis |
|  |  | 1491 | brain haemorrhage |
|  |  | 1492 | aortic aneurysm |
|  |  | 1493 | other venous/lymphatic disease |
|  |  | 1494 | varicose veins |
|  |  | 1495 | lymphoedema |
|  |  | 1496 | alpha-1 antitrypsin deficiency |
|  |  | 1583 | ischaemic stroke |
|  |  | 1584 | mitral valve disease |
|  |  | 1585 | mitral regurgitation / incompetence |
|  |  | 1586 | aortic valve disease |
|  |  | 1587 | aortic regurgitation / incompetence |
|  |  | 1588 | hypertrophic cardiomyopathy (hcm / hocm) |
|  |  | 1589 | pericarditis |
|  |  | 1590 | pericardial effusion |
|  |  | 1591 | aortic aneurysm rupture |
|  |  | 1592 | aortic dissection |
|  |  | 1593 | varicose ulcer |
| Diabetes | 20002 | 1220 | diabetes |
|  |  | 1221 | gestational diabetes |
|  |  | 1222 | type 1 diabetes |
|  |  | 1223 | type 2 diabetes |
| Endocrine | 20002 | 1224 | thyroid problem (not cancer) |
|  |  | 1225 | hyperthyroidism/thyrotoxicosis |
|  |  | 1226 | hypothyroidism/myxoedema |
|  |  | 1228 | thyroid radioablation therapy |
|  |  | 1229 | parathyroid gland problem (not cancer) |
|  |  | 1230 | parathyroid hyperplasia/adenoma |
|  |  | 1232 | disorder of adrenal gland |
|  |  | 1233 | adrenal tumour |
|  |  | 1234 | adrenocortical insufficiency/addison's disease |
|  |  | 1235 | hyperaldosteronism/conn's syndrome |
|  |  | 1236 | phaeochromocytoma |
|  |  | 1237 | disorder or pituitary gland |
|  |  | 1238 | pituitary adenoma/tumour |
|  |  | 1239 | cushings syndrome |
|  |  | 1428 | thyroiditis |
|  |  | 1429 | acromegaly |
|  |  | 1430 | hypopituitarism |
|  |  | 1431 | hyperprolactinaemia |
|  |  | 1508 | jaundice (unknown cause) |
|  |  | 1521 | diabetes insipidus |
|  |  | 1522 | grave's disease |
|  |  | 1610 | thyroid goitre |
|  |  | 1611 | hyperparathyroidism |
|  |  | 1667 | alopecia / hair loss |
|  |  | 1679 | undescended testicle |
|  |  | 1682 | benign insulinoma |
| Gastrointestinal/abdominal | 20002 | 1134 | oesophageal disorder |
|  |  | 1135 | stomach disorder |
|  |  | 1136 | liver/biliary/pancreas problem |
|  |  | 1137 | other abdominal problem |
|  |  | 1138 | gastro-oesophageal reflux (gord) / gastric reflux |
|  |  | 1139 | oesophagitis/barretts oesophagus |
|  |  | 1140 | oesophageal stricture |
|  |  | 1141 | oesophageal varicies |
|  |  | 1142 | gastric/stomach ulcers |
|  |  | 1143 | gastritis/gastric erosions |
|  |  | 1154 | irritable bowel syndrome |
|  |  | 1155 | hepatitis |
|  |  | 1156 | infective/viral hepatitis |
|  |  | 1157 | non-infective hepatitis |
|  |  | 1158 | liver failure/cirrhosis |
|  |  | 1159 | bile duct disease |
|  |  | 1160 | bile duct obstruction/ascending cholangitis |
|  |  | 1161 | gall bladder disease |
|  |  | 1162 | cholelithiasis/gall stones |
|  |  | 1163 | cholecystitis |
|  |  | 1164 | pancreatic disease |
|  |  | 1165 | pancreatitis |
|  |  | 1190 | peritonitis |
|  |  | 1191 | gastrointestinal bleeding |
|  |  | 1400 | peptic ulcer |
|  |  | 1432 | carcinoid syndrome/tumour |
|  |  | 1456 | malabsorption/coeliac disease |
|  |  | 1457 | duodenal ulcer |
|  |  | 1458 | diverticular disease/diverticulitis |
|  |  | 1459 | colitis/not crohns or ulcerative colitis |
|  |  | 1460 | rectal or colon adenoma/polyps |
|  |  | 1461 | inflammatory bowel disease |
|  |  | 1462 | crohns disease |
|  |  | 1463 | ulcerative colitis |
|  |  | 1475 | sclerosing cholangitis |
|  |  | 1501 | pyloric stenosis |
|  |  | 1503 | anal problem |
|  |  | 1504 | anal fissure |
|  |  | 1505 | haemorrhoids / piles |
|  |  | 1506 | primary biliary cirrhosis |
|  |  | 1509 | gastroenteritis/dysentry |
|  |  | 1510 | dyspepsia / indigestion |
|  |  | 1511 | abdominal hernia |
|  |  | 1512 | umbilical hernia |
|  |  | 1513 | inguinal hernia |
|  |  | 1562 | food intolerance |
|  |  | 1599 | constipation |
|  |  | 1600 | bowel / intestinal perforation |
|  |  | 1601 | bowel / intestinal infarction |
|  |  | 1602 | bowel / intestinal obstruction |
|  |  | 1603 | rectal prolapse |
|  |  | 1604 | alcoholic liver disease / alcoholic cirrhosis |
| Musculoskeletal | 20002 | 1293 | bone disorder |
|  |  | 1294 | back problem |
|  |  | 1295 | joint disorder |
|  |  | 1297 | muscle/soft tissue problem |
|  |  | 1308 | osteomyelitis |
|  |  | 1309 | osteoporosis |
|  |  | 1310 | paget's disease |
|  |  | 1311 | spine arthritis/spondylitis |
|  |  | 1312 | prolapsed disc/slipped disc |
|  |  | 1313 | ankylosing spondylitis |
|  |  | 1322 | myositis/myopathy |
|  |  | 1406 | muscle or soft tissue injuries |
|  |  | 1437 | myasthenia gravis |
|  |  | 1464 | rheumatoid arthritis |
|  |  | 1465 | osteoarthritis |
|  |  | 1466 | gout |
|  |  | 1467 | other joint disorder |
|  |  | 1474 | hiatus hernia |
|  |  | 1477 | psoriatic arthropathy |
|  |  | 1478 | cervical spondylosis |
|  |  | 1480 | dermatomyositis |
|  |  | 1481 | polymyositis |
|  |  | 1524 | spina bifida |
|  |  | 1532 | disc problem |
|  |  | 1533 | disc degeneration |
|  |  | 1534 | back pain |
|  |  | 1535 | scoliosis |
|  |  | 1536 | spinal stenosis |
|  |  | 1537 | joint pain |
|  |  | 1538 | arthritis (nos) |
|  |  | 1540 | plantar fascitis |
|  |  | 1541 | carpal tunnel syndrome |
|  |  | 1542 | fibromyalgia |
|  |  | 1544 | dupuytren's contracture |
|  |  | 1545 | neck problem/injury |
|  |  | 1617 | osteopenia |
|  |  | 1618 | soft tissue inflammation |
|  |  | 1619 | tendonitis / tendinitis / tenosynovitis |
|  |  | 1620 | bursitis |
|  |  | 1621 | synovitis |
|  |  | 1622 | epicondylitis |
|  |  | 1623 | tennis elbow / lateral epicondylitis |
|  |  | 1624 | housemaid's knee (prepatellar bursitis) |
|  |  | 1625 | cellulitis |
| Neurological | 20002 | 1240 | neurological injury/trauma |
|  |  | 1244 | infection of nervous system |
|  |  | 1245 | brain abscess/intracranial abscess |
|  |  | 1246 | encephalitis |
|  |  | 1247 | meningitis |
|  |  | 1248 | spinal abscess |
|  |  | 1249 | cranial nerve problem/palsy |
|  |  | 1250 | bell's palsy/facial nerve palsy |
|  |  | 1251 | spinal cord disorder |
|  |  | 1252 | paraplegia |
|  |  | 1254 | peripheral nerve disorder |
|  |  | 1255 | peripheral neuropathy |
|  |  | 1256 | acute infective polyneuritis/guillain-barre syndrome |
|  |  | 1257 | trapped nerve/compressed nerve |
|  |  | 1258 | chronic/degenerative neurological problem |
|  |  | 1259 | motor neurone disease |
|  |  | 1260 | myasthenia gravis |
|  |  | 1261 | multiple sclerosis |
|  |  | 1262 | parkinsons disease |
|  |  | 1263 | dementia/alzheimers/cognitive impairment |
|  |  | 1264 | epilepsy |
|  |  | 1265 | migraine |
|  |  | 1266 | head injury |
|  |  | 1267 | spinal injury |
|  |  | 1394 | peripheral nerve injury |
|  |  | 1397 | other demyelinating disease (not multiple sclerosis) |
|  |  | 1415 | ear/vestibular disorder |
|  |  | 1420 | otosclerosis |
|  |  | 1421 | meniere's disease |
|  |  | 1425 | cerebral aneurysm |
|  |  | 1433 | cerebral palsy |
|  |  | 1434 | other neurological problem |
|  |  | 1436 | headaches (not migraine) |
|  |  | 1468 | diabetic neuropathy/ulcers |
|  |  | 1476 | sciatica |
|  |  | 1499 | labyrinthitis |
|  |  | 1500 | vertigo |
|  |  | 1523 | trigemminal neuralgia |
|  |  | 1525 | benign / essential tremor |
|  |  | 1561 | raynaud's phenomenon/disease |
|  |  | 1597 | tinnitus / tiniitis |
|  |  | 1616 | insomnia |
|  |  | 1659 | meningioma / benign meningeal tumour |
|  |  | 1683 | benign neuroma |
| Psychological | 20002 | 1243 | psychological/psychiatric problem |
|  |  | 1286 | depression |
|  |  | 1287 | anxiety/panic attacks |
|  |  | 1288 | nervous breakdown |
|  |  | 1289 | schizophrenia |
|  |  | 1290 | deliberate self-harm/suicide attempt |
|  |  | 1291 | mania/bipolar disorder/manic depression |
|  |  | 1408 | alcohol dependency |
|  |  | 1409 | opioid dependency |
|  |  | 1410 | other substance abuse/dependency |
|  |  | 1469 | post-traumatic stress disorder |
|  |  | 1470 | anorexia/bulimia/other eating disorder |
|  |  | 1531 | post-natal depression |
|  |  | 1614 | stress |
|  |  | 1615 | obsessive compulsive disorder (ocd) |
| Renal | 20002 | 1192 | renal/kidney failure |
|  |  | 1193 | renal failure requiring dialysis |
|  |  | 1194 | renal failure not requiring dialysis |
|  |  | 1196 | urinary tract infection/kidney infection |
|  |  | 1197 | kidney stone/ureter stone/bladder stone |
|  |  | 1200 | ureteric obstruction/hydronephrosis |
|  |  | 1201 | bladder problem (not cancer) |
|  |  | 1202 | urinary frequency / incontinence |
|  |  | 1207 | prostate problem (not cancer) |
|  |  | 1210 | scrotal problem (not cancer) |
|  |  | 1214 | testicular problems (not cancer) |
|  |  | 1396 | enlarged prostate |
|  |  | 1404 | male infertility |
|  |  | 1405 | other renal/kidney problem |
|  |  | 1427 | polycystic kidney |
|  |  | 1514 | cystitis |
|  |  | 1515 | pyelonephritis |
|  |  | 1516 | bph / benign prostatic hypertrophy |
|  |  | 1517 | prostatitis |
|  |  | 1518 | erectile dysfunction / impotence |
|  |  | 1519 | kidney nephropathy |
|  |  | 1520 | iga nephropathy |
|  |  | 1607 | diabetic nephropathy |
|  |  | 1608 | nephritis |
|  |  | 1609 | glomerulnephritis |
| Respiratory | 20002 | 1111 | asthma |
|  |  | 1112 | chronic obstructive airways disease/copd |
|  |  | 1113 | emphysema/chronic bronchitis |
|  |  | 1114 | bronchiectasis |
|  |  | 1115 | interstitial lung disease |
|  |  | 1117 | other respiratory problems |
|  |  | 1120 | asbestosis |
|  |  | 1121 | pulmonary fibrosis |
|  |  | 1122 | fibrosing alveolitis/unspecified alveolitis |
|  |  | 1123 | sleep apnoea |
|  |  | 1124 | respiratory failure |
|  |  | 1125 | pleurisy |
|  |  | 1126 | spontaneous pneumothorax/recurrent pneumothorax |
|  |  | 1398 | pneumonia |
|  |  | 1411 | lung abscess |
|  |  | 1412 | bronchitis |
|  |  | 1413 | nasal/sinus disorder |
|  |  | 1414 | throat or larynx disorder |
|  |  | 1416 | chronic sinusitis |
|  |  | 1417 | nasal polyps |
|  |  | 1418 | chronic laryngitis |
|  |  | 1419 | vocal cord polyp |
|  |  | 1472 | emphysema |
|  |  | 1486 | sick sinus syndrome |
|  |  | 1497 | pneumothorax |
|  |  | 1498 | empyema |
|  |  | 1595 | pleural plaques (not known asbestosis) |
|  |  | 1596 | pleural effusion |
| Any Cancer | 20001 | All codes | Report of any cancer type |

Supplementary Table 2. Mortality risk for six sleep disturbance-diabetes groups.

|  |  | Age & Sex Adjusted | | Fully Adjusted | |
| --- | --- | --- | --- | --- | --- |
| All-cause mortality | | | | | |
| Diabetes Status | Sleep Disturbances | HR (95% CI) | P value | HR (95% CI) | P value |
| No Diabetes | Never | reference |  | reference |  |
|  | Sometimes | 1.06 (1.02, 1.10) | .004 | 1.02 (0.98, 1.06) | .36 |
|  | Usually | 1.26 (1.21, 1.32) | <.001 | 1.12 (1.08, 1.17) | <.001 |
| Diabetes | Never | 1.95 (1.78, 2.15) | <.001 | 1.60 (1.45, 1.77) | <.001 |
|  | Sometimes | 2.18 (2.04, 2.33) | <.001 | 1.73 (1.61, 1.86) | <.001 |
|  | Usually | 2.75 (2.57, 2.94) | <.001 | 1.90 (1.76, 2.05) | <.001 |
| CVD Mortality | | | | | |
|  |  | Age & Sex Adjusted | | Fully Adjusted | |
| Diabetes Status | Sleep Disturbances | HR (95% CI) | P value | HR (95% CI) | P value |
| No Diabetes | Never | reference |  | reference |  |
|  | Sometimes | 1.04 (0.95, 1.13) | .43 | 0.97 (0.89, 1.06) | .48 |
|  | Usually | 1.26 (1.15, 1.38) | <.001 | 1.03 (0.94, 1.14) | .51 |
| Diabetes | Never | 3.31 (2.82, 3.89) | <.001 | 2.10 (1.77, 2.50) | <.001 |
|  | Sometimes | 3.28 (2.88, 3.72) | <.001 | 1.95 (1.70, 2.24) | <.001 |
|  | Usually | 4.20 (3.70, 4.77) | <.001 | 2.06 (1.78, 2.38) | <.001 |
